# Interpersonal context and desired emotional closeness in neural response to negative visual stimuli: Preliminary findings

**DOI:** 10.1002/brb3.2438

**Published:** 2021-12-07

**Authors:** Luis E. Flores, Gabriela Alarcón, Kristen L. Eckstrand, Morgan Lindenmuth, Erika E. Forbes

**Affiliations:** ^1^ Department of Psychology Queen's University Kingston Canada; ^2^ Department of Psychiatry University of Pittsburgh Pittsburgh Pennsylvania USA

**Keywords:** desired emotional closeness, emotion, fMRI, interpersonal, social regulation

## Abstract

**Introduction:**

Emotions typically emerge in interpersonal contexts, but the neural circuitry involved remains insufficiently understood. Two key features of interpersonal contexts are interpersonal interactions (e.g., supportive physical touch serving as a form of social regulation) and interpersonal traits. Social regulation research has predominately focused on fear by using physical threat (i.e., electric shock) as the stimulus. Given that social regulation helps with various negative emotions in the real world, using visual stimuli that elicit negative emotions more broadly would also be beneficial. Differing from trait loneliness—which is related to lower recruitment of social circuitry in negative socioaffective contexts—trait desired emotional closeness is related to adaptive outcomes and may demonstrate an opposite pattern. This study investigated the roles of social regulation and desired emotional closeness in neural response to aversive social images.

**Methods:**

Ten pairs of typically developing emerging adult friends (N = 20; ages 18–25) completed a functional magnetic resonance imaging (fMRI) handholding task. Each friend viewed negative and neutral social images in the scanner under two conditions: (a) holding their friend's hand and (b) having their friend in the room.

**Results:**

Handholding attenuated response to aversive social images in a region implicated in emotion and inhibitory control (right dorsal striatum/anterior insula/ventrolateral prefrontal cortex). Desired emotional closeness was positively associated with response to aversive social images (in the no handholding condition) in self and social processing (right ventral posterior cingulate cortex) and somatosensory regions (right postcentral gyrus).

**Discussion:**

These findings extend previous research on the roles of interpersonal behaviors and tendencies in neural response to aversive stimuli.

## Graphical abstract

Most emotion research focuses on intrapersonal factors despite the critical role of interpersonal context; thus, the neural circuitry involved in emotion that arises in interpersonal contexts remains insufficiently understood. Two key features of interpersonal contexts are interpersonal interactions (e.g., handholding as a form of supportive physical touch) and interpersonal traits (e.g., desired emotional closeness). In a functional magnetic resonance imaging (fMRI) study, we found that handholding attenuated neural response to aversive social images in a region implicated in emotion and inhibitory control and that desired emotional closeness was positively associated with neural response to aversive social images in self and social processing and somatosensory regions.

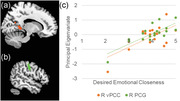

## 1 INTRODUCTION

Most emotion research focuses on intrapersonal factors, such as intrapersonal emotion regulation strategies (e.g., cognitive reappraisal); however, the interpersonal context (e.g., supportive physical touch serving as a form of social regulation) also plays a critical but poorly understood role. Two key features of the interpersonal context in which emotions develop are (1) interpersonal interactions (e.g., whether a close other helps to regulate an emotional response) and (2) a person's interpersonal traits (i.e., personality characteristics related to an individual's patterns of interacting with other people). Recently, research and frameworks for understanding social regulation have been emerging (e.g., Butler & Randall, 2013; Coan & Maresh, 2014; Reeck et al., 2016; Zaki & Williams, 2013). Coan's (2008) social baseline theory explains that the mere presence of another person reduces negative emotional responses by sharing the load of threats and decreasing associated risks. It posits that the human brain has evolved to operate optimally in the presence of other people and that more cognitive resources (e.g., those needed for intrapersonal strategies) are typically required to regulate emotions when a person is alone (Coan, 2008). However, further elucidation of the neural circuitry involved is needed, as previous research provided valuable knowledge about attenuation of threat responding but did not include visual stimuli or a range of negative stimuli (e.g., stimuli that tend to elicit sadness, disgust, fear, and/or anger). Given the importance of visual input to affective responding (Barrett & Bar, 2009) and the potential for social regulation across types of emotion, it is critical to examine the social regulation of neural reponse under more general negative emotional contexts. Considering that a key component of social baseline theory is the presence of other people, valuing emotionally intimate interactions may be an interpersonal trait that also plays a role in reactivity to aversive situations. Examining both social regulation and interpersonal traits could help clarify different components of the role of interpersonal context in emotion.

### 1.1 Features of interpersonal context

#### 1.1.1 Interpersonal interactions: Social regulation

Physical touch is a ubiqutious form of social regulation that provides a powerful signal of support (Fredrickson, 2013). For example, people often hug when someone feels sad or hold hands when someone is about to receive potentially exciting or disappointing news (e.g., the outcome of a competition). More broadly, physical touch plays a role in many interpersonal functions, including attachment, intimacy, pleasure, compliance, power, and emotional communication (Hertenstein et al., 2006). Physical touch is thought to be used more than verbal communication, particularly when under stress, due to its earlier development in both human evolution and modern human development (Burgoon et al., 2016; Field, 2019). In fact, physical touch is commonly used for soothing and reconciliation after conflict in nonhuman primates (de Waal, 2000). Due to its ubiquity and effectiveness, handholding has primarily been used to examine the social regulation of neural response. When used during the anticipation of electric shock, handholding attenuates response in regions of the salience network (e.g., anterior cingulate cortex, striatum) and cognitive control network (e.g., dorsolateral prefrontal cortex, DLPFC; Coan et al., 2013, 2006). The salience network is implicated in emotion and detecting threats (Menon, 2011). The cognitive control network is involved in high‐level cognitive processes, including those recruited for cognitive/effortful emotion regulation (Niendam et al., 2012). Social baseline theory explains that this pattern of findings may be related to an attenuation of neural threat response that requires fewer cognitive resources than those needed for cognitive/effortful emotion regulation (Coan, 2008).

It is currently unclear how the social regulation attenuates neural response to negative visual stimuli that elicit a broad range of negative emotions, including social stimuli that involve seeing people in distressing situations. Previous studies have focused on handholding during the nonsocial threat of electric shock (Coan et al., 2013, 2006). An advantage of using the threat of electric shock as the negative stimulus is that it primarly elicits a specific emotion—fear. Physical touch in the real world, however, is broadly used for a variety of negative emotions, including sadness. Therefore, it would also be beneficial to use stimuli that elicit a broad range of negative emotions, such as images depicting people in situations involving loss, danger, and disease.

Although the use of physical touch in an aversive emotional context that elicits a broad range of negative emotions (e.g., validated stimulus sets, such as the International Affective Picture System [IAPS], that are intended to elicit negative affect rather than specific negative emotions; Lang et al., 2008) has not been investigated at the neural level, it has been examined at the behavioral level. Physical touch has been found to attenuate self‐reported negative emotion, negative facial expressivity, and negative emotional memory (Flores & Berenbaum, 2012, 2017a). There is also evidence that physical touch helps improve the updating of negative contents (i.e., negative IAPS images) in working memory (Flores & Berenbaum, 2017b). Thus, there is behavioral evidence that physical touch can effectively serve as a form of social regulation in a broadly negative emotional context.

Social regulation attenuates a greater number of brain regions implicated in emotion and cognitive control when conducted by someone emotionally close to the individual (e.g., a spouse) compared to when it is conducted by a stranger (Coan et al., 2013, 2006). Thus, degree of closeness matters, and it may be related to greater availability to share the load of the threat. It is unclear whether degree of physical presence (e.g., being physically nearby vs. touching) also matters. Although both physical proximity and physical touch may serve as forms of social regulation, physical touch may provide a stronger indication than physical proximity that someone is present to share the load of a threat.

#### 1.1.2 Interpersonal traits: Desired emotional closeness

Desired emotional closeness refers to the extent of valuing and wanting emotionally intimate experiences with others, such as physical and verbal affection, self‐disclosure of personal thoughts, emotional support, and feeling emotionally close (Flores & Berenbaum, 2012, 2014). Behavioral studies suggest that trait desired emotional closeness plays a role in affective processes. For example, a high level of desired emotional closeness is related to greater effectiveness of social regulation, as measured by facial expressivity in response to negative images (Flores & Berenbaum, 2012). Also, although social regulation improves the ability to remove irrelevent negative contents (i.e., images) from working memory among individuals with higher desired emotional closeness, social regulation worsens this ability among those with lower desire (Flores & Berenbaum, 2017b). Lastly, having emotionally intimate experiences (i.e., perceived emotional closeness) predicts lower psychological distress the next day among individuals with higher desired emotional closeness but not among those with lower desire (Flores & Berenbaum, 2014).

Although the role of desired emotional closeness in neural response to negative stimuli has not yet been examined, a related interpersonal trait—loneliness—is associated with greater activation of the visual cortex but lower activation in social circuitry (i.e., temporoparietal junction; TPJ) in response to negative social images compared with negative nonsocial images (Cacioppo et al., 2009). Similar to desired emotional closeness, loneliness refers to wanting social connection. However, in contrast to desired emotional closeness, loneliness also captures psychological distress about having perceived difficulty obtaining social connection. Notably, whereas desired emotional closeness is negatively associated with depression and positively associated with perceived emotional closeness (Flores & Berenbaum, 2014), loneliness is positively related to depression (Cacioppo et al., 2006) and negatively associated with perceived emotional closeness (Hall et al., 2020). Desired emotional closeness may also demonstrate an opposite pattern in social circuitry (i.e., greater activation) in response to negative social images with relevance to adaptive functioning.

### 1.2 Emerging adulthood

Emerging adulthood (ages 18–25 years) is characterized by challenging social role transitions (e.g., moving away from home, living independently, and attending university) that carry more responsibility but less structure than what is experienced during childhood and adolescence. This is an important developmental period to examine social regulation for several reasons. First, social interactions are particularly salient and desired among emerging adults (Arnett, 2007). Second, emerging adulthood is a period of social instability with social networks changing at a rapid pace (Shanahan, 2000). Third, emerging adults are learning how to manage adult relationships while undergoing neurodevelopment in social circuitry (Taber‐Thomas & Pérez‐Edgar, 2015). Fourth, emerging adults continue to exhibit neurodevelopment related to emotion regulation (Taber‐Thomas & Pérez‐Edgar, 2015). In fact, emotion regulation ability increases over the life span (Charles & Carstensen, 2014). Thus, emerging adulthood is the adult developmental period with the lowest emotion regulation ability. Fifth, emerging adulthood has the highest rate of depression among adult age groups (SAMHSA, 2018).

### 1.3 Present study

To inform and encourage further research on interpersonal context in neural response to negative visual stimuli, the present study examined neural response to negative social images among emerging adult friends in a functional magnetic resonance imaging (fMRI) handholding task. Our emphasis on neural response rather than subjective response is informed by social baseline theory positing that the conservation of cognitive resources is a distinguishing factor of social regulation compared to most intrapersonal regulation strategies (Coan, 2008). The first aim was to examine the main effect of social regulation on neural response to a set of social visual stimuli that elicit a broad range of negative emotions. To achieve this, participants viewed IAPS images (Lang et al., 2008) of people in sad or horrifying situations while having a friend holding their hand (social regulation condition) versus having a friend physically nearby (less active “control” physical proximity condition). We hypothesized that social regulation would attenuate regions of the salience and cognitive control networks, as found in social regulation studies using threat of electric shock (Coan et al., 2013, 2006). The second aim of the study was to examine whether trait desired emotional closeness was associated with neural response to negative social images. We hypothesized that trait desired emotional closeness would be associated with greater activation of social circuitry when viewing negative social images in the “control” physical proximity condition. Given previous behavioral findings on the moderating role of trait desired emotional closeness on social regulation (e.g., Flores & Berenbaum, 2012, 2014, 2017b), we would expect trait desired emotional closeness to moderate the social regulation of neural response to negative images. However, we did not include this as an aim of the study due to the modest sample size of the study.

## 2 METHODS

### 2.1 Participants

The diverse sample of participants included 20 typically developing emerging adults (10 pairs of self‐identified close friends) between 18 and 25 years old (*M =* 21.20, *SD* = 1.74 years; 55% women; 0% Hispanic/Latinx; 35% White, 30% Black, 25% Asian, 10% other or multiracial). Seven pairs were same‐gender friends (four pairs were women and three pairs were men) and three pairs were mixed‐gender friends. Pairs were of any gender combination due to the assumption that degree of closeness would be more influential to the effectiveness of the social regulation of neural response than gender. Inclusion and exclusion criteria were screened by phone to recruit a sample with no history of psychiatric or serious medical problems, heavy alcohol or cannabis use, regular use of tobacco or other illicit substances, current use of stimulant medication or psychotropic medication within the past 6 weeks, or presence of MRI contraindications. Psychiatric history was screened using the following question: “Have you ever been diagnosed with or do you suspect you may have ever had a mental or behavioral disorder, such as depression, anxiety, bipolar disorder, schizophrenia, autism spectrum disorder, or ADHD? If so, what was the disorder?”

The sample size of 20 participants was determined with the intention of providing preliminary findings with large effect sizes. We conducted a power analysis using G*Power 3.1 (Faul et al., 2009) for each of the two study aims. For the first study aim comparing neural responses to negative images in physical proximity and handholding conditions, we found that our sample size provides approximately 80% power to detect a large effect size (*d* = 0.70) in a paired‐sample *t*‐test. For the second study aim examining the association between desired emotional closeness and neural response to negative images in the physical proximity condition, we found that our sample size provides approximately 70% power to detect a large effect size (*f^2^ =* 0.35) in a linear regression with one predictor. Expecting large effect sizes was reasonable given that previous fMRI studies examining handholding have found large effects with comparable sample sizes (e.g., Coan et al., 2013, 2006).

### 2.2 Measures

#### 2.2.1 Desired emotional closeness

Participants completed a 20‐item self‐report brief version of the Emotional Closeness Questionnaire (Brief ECQ; Flores & Berenbaum, 2012, 2014). The desired emotional closeness subscale within the Brief ECQ included 10 items that assess the extent to which respondents want to engage in different types of emotionally intimate experiences with close others on a five‐point Likert scale (1 = not at all; 5 = extremely; *M* = 3.97, *SD* = 0.70, range = 2.10–5.00). The distribution of responses for the subscale was typical for a nonclinical sample (Flores & Berenbaum, 2012). Although women scored slightly higher than men on desired emotional closeness (4.22 vs. 3.66), this difference fell short of statistical significance, *t*(18) = −1.95, *p* = .066. Age was not significantly correlated with desired emotional closeness, *r* = 0.05, *p* = .830. One mild outlier was identified in a box‐and‐whisker plot of desired emotional closeness (i.e., value was more than 1.5 times the interquartile range away from the mean). This outlier was kept unchanged in analyses given that significant findings remained when removing it in follow‐up analyses (see Section 3). The subscale's internal consistency value in this sample was high (*α =* .91).

#### 2.2.2 Social regulation of neural response to negative visual stimuli

Participants completed an adapted version of the handholding fMRI task developed by Coan et al. (2006). Coan et al. (2013, 2006) have found that holding the hand of a spouse, stranger, or friend each attenuates neural response to negative stimuli, despite doing so to varying degrees. Whereas the original task used the threat of electric shock to elicit negative emotion, this adapted version used aversive images. A behavioral version of this type of adaptation has previously been used effectively while holding the hand of a stranger (Flores & Berenbaum, 2012, 2017a, 2017b). In the present study, for each pair of participants, one friend went first in the scanner to view negative and neutral social images under two conditions: (a) while holding their friend's hand (social regulation condition) and (b) while just having their friend in the room (less active “control” physical proximity condition). Afterward, the friends switched roles; friends could not view the images when outside the scanner. Handholding condition order was counter‐balanced. Each condition included four picture‐valence blocks (two negative and two neutral blocks), which consisted of either negative or neutral IAPS images (Lang et al., 2008). Participants completed all four picture‐valence blocks within one condition before transitioning to the next condition. Within each condition, the order of picture‐valence blocks and pictures within the blocks was randomized, such that the order of blocks and pictures varied by participant. All pictures were social in nature, such that they had people in them (e.g., neutral pictures: people having a conversation, neutral faces; negative pictures: angry faces, bloody bodies). Negative IAPS pictures are designed to elicit a broad range of negative emotions rather than a specific emotion. Although neutral and negative pictures were not matched for perceptual features, there were no statistically significant differences in brightness or luminance contrast between neutral and negative pictures. Supporting material includes the list of images. Within each block, participants viewed 12 pictures for 3 s each with a jittered intertrial fixation screen between stimuli (1, 3, 5, or 7 s). Each block contained two stimuli of the other type (e.g., a negative block contained two neutral images) to reduce habituation and predictability. Participants then rated their negative mood and arousal on a five‐point Likert scale version (1 = no negative mood or arousal; 5 = extremely high negative mood or arousal) of the pictorial Self‐Assessment Mannequin (Bradley & Lang, 1994), followed by an interblock interval of 8 s. The task lasted about 15 min per participant. We calculated descriptive statistics for self‐reported negative mood during the neutral (physical proximity: *M* = 1.53, *SD* = 0.80; handholding: *M* = 1.61, *SD* = 0.97) and negative blocks (physical proximity: *M* = 3.15, *SD* = 1.11; handholding: *M* = 3.10, *SD* = 1.05), and for self‐reported perceived bodily arousal during the neutral (physical proximity: *M* = 1.44, SD = 0.75; handholding: *M* = 1.53, SD = 0.78) and negative blocks (physical proximity: *M* = 2.25, *SD* = 1.13; handholding: *M* = 2.31, *SD* = 1.28). After the task, participants rated how comfortable and distracting they found holding their friend's hand on a five‐point scale (1 *=* not at all, 5 = extremely; comfortable: *M* = 4.15, *SD* *=* 0.99; distracting: *M =* 1.45, *SD* = 0.83). These scores suggest that participants generally found the handholding condition to be comfortable and not distracting. These scores were not significantly associated with attenuation of neural response to negative (vs. neutral) images by handholding.

### 2.3 fMRI acquisition and preprocessing

We used a Siemens 3T Trio scanner at the University of Pittsburgh Magnetic Resonance Research Center (MRRC). MPRAGE structural images were acquired with high‐resolution T1‐weighted images with 1‐mm isometric voxels (TR/TE/TI/flip angle = 1500 ms/3.19 ms/800 ms/8 degrees; FOV = 256 mm × 256 mm; 1.00‐mm slice; 176 slices; 256 × 256 matrix). Functional blood‐oxygen‐level‐dependent (BOLD) images were acquired using gradient echo planar imaging (EPI) with a simultaneous multi‐slice sequence (multi‐band = 3; radiofrequency pulse duration = 3840 μs); 18 oblique axial slices (2.3 mm thick, 0 mm gap) oriented to the AC‐PC line (TR/TE/flip angle = 1500 ms/30 ms/58 degrees; FOV = 220 mm × 220 mm; matrix = 96 × 96). A reference EPI scan acquired prior to fMRI data collection was visually inspected for artifacts and signal quality.

We performed fMRI analyses using Statistical Parametric Mapping software, version 12 (SPM12; http://www.fil.ion.ucl.ac.uk/spm/software/spm12/). Images for each subject were realigned, motion‐corrected, and high‐pass temporally filtered with a cutoff of 128 s. High‐motion volumes (≥2 mm) were removed using Artifact Detection Tool (ART; https://www.nitrc.org/projects/artifact_detect). The number of motion outliers did not significantly differ by social regulation conditions, *t*(19) = 0.41, *p* = .687, or valence blocks, *t*(19) = 0.57, *p* = .578. The mean functional image was coregistered with the high‐resolution 3D anatomic image, normalized to Montreal Neurological Institute (MNI) space, and spatially smoothed (Gaussian kernel 6.0 mm full‐width half‐maximum).

### 2.4 Data analytic strategy

Level 1 contrasts compared neural response to stimuli in the negative versus neutral blocks (negative > neutral) within each condition. These individual level analyses included noninterest periods (i.e., interblock intervals, ratings periods, break after each condition) and motion parameters as regressors of noninterest.

We conducted a whole‐brain full factorial model using the above Level 1 contrasts as the dependent variable to test each hypothesis. The model included one within‐person two‐level factor of condition (physical proximity vs. handholding) and three covariates (gender, age, and desired emotional closeness). Gender was included as a covariate as there is some evidence of women benefiting more than men from social regulation (Flores & Berenbaum, 2012). In addition, women may be more comfortable holding hands with a friend. Age was included given that there is continued neurodevelopment in regions related to emotion and social processing during emerging adulthood (Taber‐Thomas & Pérez‐Edgar, 2015). First, to determine sample‐wide neural response to negative images and to ensure that the negative images engaged emotion‐related circuitry, we examined the Level 1 contrast comparing blocks (negative > neutral) in the physical proximity condition. Second, social regulation of neural response (i.e., attenuation of neural response by handholding) was demonstrated by the main effect of condition, which compared neural response to negative images (level 1 contrast: negative > neutral blocks) in the physical proximity condition with neural response to negative images (level 1 contrast: negative > neutral blocks) in the handholding condition (level 2 contrast: handholding < physical proximity and handholding > physical proximity). Third, the role of desired emotional closeness in neural response to negative visual stimuli was examined by testing the association between trait desired emotional closeness and neural response to negative images in the physical proximity condition. Tests were thresholded at voxel‐level *p* < .001 and cluster‐level false discovery rate (FDR) *p* < .05.

Two multilevel models were conducted to examine the effect of negative versus neutral images and social regulation on self‐reported mood and perceived bodily arousal. We used the MIXED procedure of the SAS 9.4 software. We included a repeated statement for condition and used robust standard errors. Valence (dummy‐coded; negative = 1), condition (dummy‐coded; handholding = 1), and a valence × condition interaction (which is important to examine a significant effect of social regulation) were included as predictors and self‐reported negative mood and perceived bodily arousal were included as outcome variables, respectively. Given that participants were pairs of friends, we checked the intraclass correlation coefficients (ICC) to examine how consistent neural and subjective responses were within friend pairs. As expected, friend pairs exhibited generally low ICCs in neural response (−0.13), self‐report mood (0.13), and self‐report perceived bodily arousal (0.31). Thus, there appears to be low consistency between friends, which suggests low need to account for friendship pairs in statistical models.

## 3 RESULTS

### 3.1 Social regulation of neural response to aversive images

**FIGURE 1:**
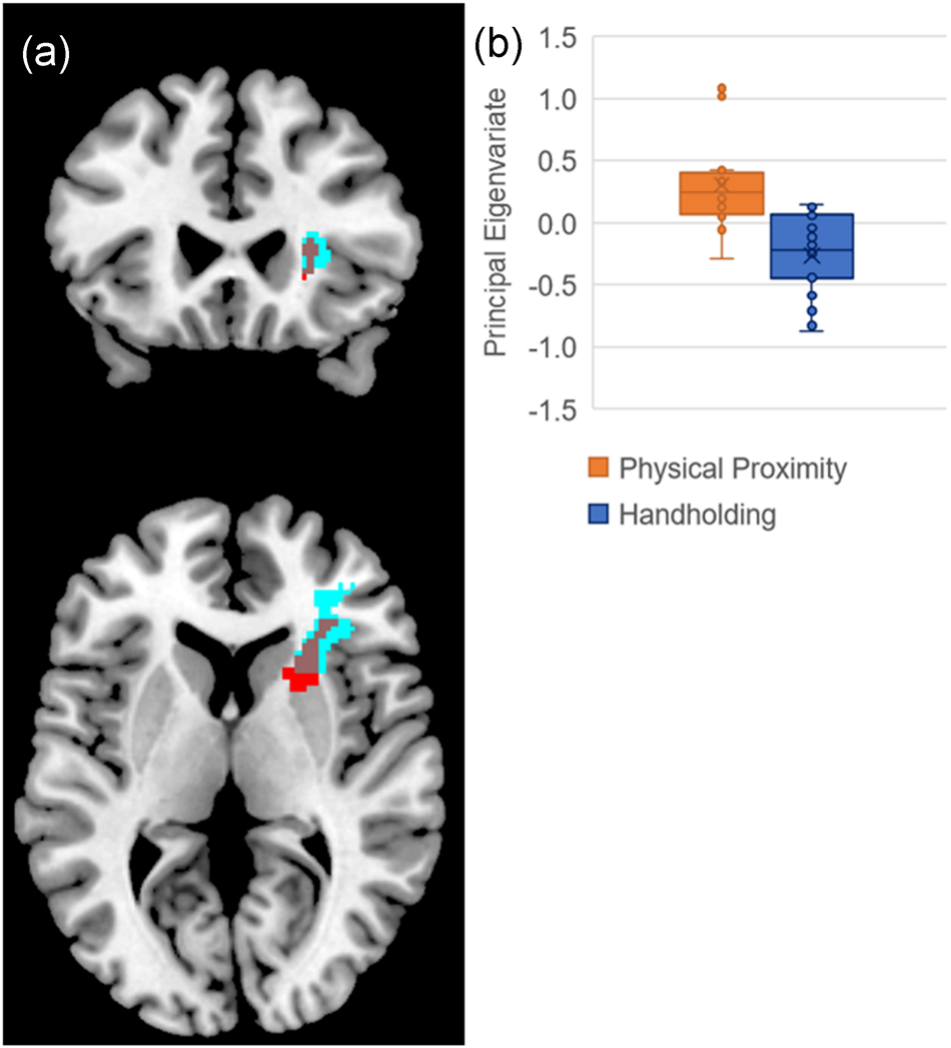
Social regulation of neural response to negative images. (a) Red represents the cluster (right dorsal striatum/ventrolateral prefrontal cortex [VLPFC]) showing response to negative visual stimuli (compared with neutral stimuli) in the physical proximity condition; blue represents the cluster (right dorsal striatum/anterior insula/VLPFC) showing attenuation to negative stimuli (compared with neutral stimuli) by social regulation (handholding condition < physical proximity condition); brown voxels represent the overlap between the two (i.e., voxels that demonstrate both neural response to negative stimuli and attenuation of neural response to negative stimuli by social regulation). Image includes coronal (Y = 25) and axial views (Z = 6). (b) Box and whisker plots of principal eigenvariates in physical proximity and handholding conditions for the cluster associated with attenuation of neural response to negative stimuli by social regulation (i.e., cluster that includes both blue and brown voxels in Figure 1a)

Based on the full factorial whole‐brain analyses (see Table 1), participants demonstrated a significant neural response to negative versus neutral visual stimuli during the physical proximity condition in the right dorsal striatum/ventrolateral prefrontal cortex (VLPFC). As expected, there was not a significant neural response to negative versus neutral visual stimuli during the handholding condition. Participants also demonstrated attenuation of neural response (level 1 contrasts: negative > neutral blocks) in an overlapping region during handholding compared with the physical proximity condition (level 2 contrast: handholding condition < physical proximity condition; see Figure 1). This region included portions of the right dorsal striatum, anterior insula, and VLPFC. No regions had a significant response to neutral versus negative stimuli within the physical proximity condition or in the handholding > physical proximity contrast. A box‐and‐whisker plot (see Figure 1b) revealed three outliers in the physical proximity condition. As a precaution, we used the extracted principal eigenvariates (negative > neutral blocks) to conduct two follow‐up paired sample *t*‐tests (physical proximity vs. handholding conditions) that: (1) excluded these outliers and (2) excluded two participants who revealed after the session that they were in a romantic relationship. The difference between conditions in this brain region remained significant. We also reran the full factorial model to test differences between friend pairs who were same versus different gender (differences were not significant) and to test order effects. Neither social regulation condition order (i.e., doing physical proximity or handholding condition first) nor “role order” within friendship pairs (i.e., being in scanner or being “handholder” first) significantly interacted with the social regulation condition. There were no brain areas with significant gender or age effects in response to negative images in either condition or in attenuation of response by handholding.

**TABLE 1:** Brain regions that demonstrated response to negative visual stimuli, attenuation by social regulation, and positive associations between response to negative visual stimuli and trait desired emotional closeness

Brain region	Number of voxels in cluster	Max. T‐score at peak voxel	MNI coordinates of peak voxel (x, y, z)		
Main effect of negative blocks in physical proximity condition					
R dorsal striatum/VLPFC, BA 45	216	5.34	24	18	10
Main effect of handholding condition					
R dorsal striatum/anterior insula/VLPFC, BA 45	318	5.51	26	22	6
Positive association between response to negative blocks in physical proximity condition and trait desired emotional closeness					
R vPCC, BA 23/30	202	4.34	16	−48	6
R postcentral gyrus, BA 3/4	177	4.86	66	−16	42

*Note*. Results were thresholded at *p* < .001 at the voxel‐level. The clusters reported here are significant using FDR cluster corrections (*p* < .05). Abbreviations: BA, Brodmann Area; MNI, Montreal Neurological Institute; R, right; VLPFC, ventrolateral prefrontal cortex; vPCC, ventral posterior cingulate cortex.

### 3.2 Association between trait desired emotional closeness and neural response to negative visual stimuli

**FIGURE 2:**
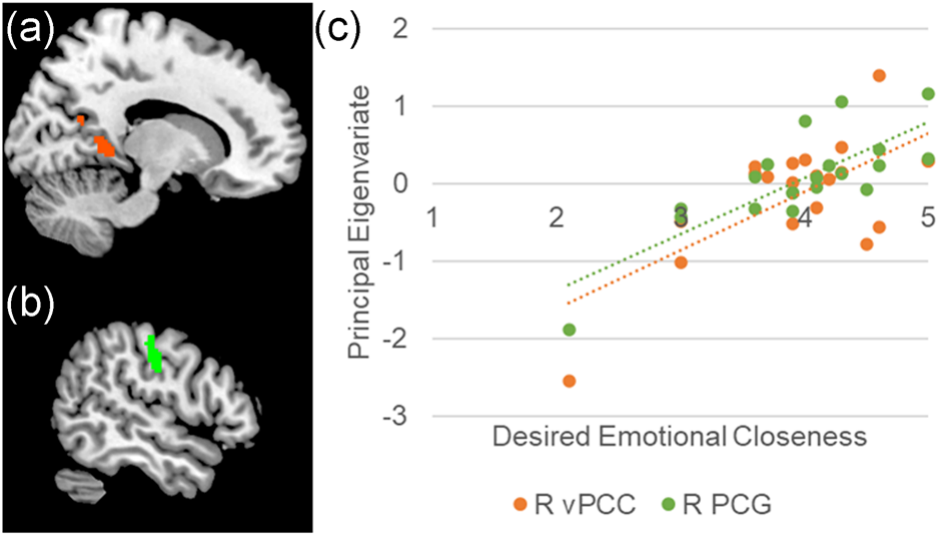
Association between neural response to negative visual stimuli in physical proximity condition and trait desired emotional closeness. (a–b) Brain regions in which the response to negative visual stimuli within the physical proximity condition (negative blocks > neutral blocks) is associated with trait desired emotional closeness: A cluster including the right ventral posterior cingulate cortex (vPCC) (Figure 2a; X = 14) and a cluster including the right postcentral gyrus (Figure 2b; X = 52). (c) Scatterplot of associations between response to negative visual stimuli within the physical proximity condition and trait desired emotional closeness for each cluster. Abbreviations: R, right; vPCC, ventral posterior cingulate cortex; PCG, postcentral gyrus

Two clusters demonstrated significant positive associations between trait desired emotional closeness and response to negative visual stimuli in the physical proximity condition (see Figure 2). One cluster includes portions of the right ventral posterior cingulate cortex (vPCC). The other cluster includes portions of the right postcentral gyrus (PCG). As a precaution, we used the extracted principal eigenvariates (negative > neutral blocks in physical proximity condition) to conduct follow‐up bivariate correlations for each cluster that: (1) excluded one desired emotional closeness outlier and (2) excluded two participants who revealed after the session that they were in a romantic relationship. The bivariate correlations remained significant for each cluster. As expected, trait desired emotional closeness was not significantly associated with neural response to negative visual stimuli in the handholding condition.

### 3.3 Subjective response to aversive social images and social regulation

The main effect of valence (i.e., subjective response to aversive images) on self‐reported negative mood (coefficient estimate [standard error; SE] = 1.64 [0.12], *t*[19.5] = 13.47, *p* < .001) and self‐reported perceived bodily arousal (coefficient estimate [SE] = 0.84 [0.16], *t*[19.4] = 5.37, *p* < .001) were significant, such that they were higher for negative image blocks compared with neutral image blocks. The valence × condition interaction (i.e., subjective response to social regulation) fell short of significance for self‐reported negative mood (coefficient estimate [standard error; SE] = −0.29 [0.16], *t*[15.3] = −1.85, *p* = .08), and was not significant for self‐reported perceived bodily arousal (coefficient estimate [SE] = −0.01 [0.19], *t*[19.2] = −0.04, *p* = .97).

## 4 DISCUSSION

The present study demonstrated the role of interpersonal context in neural response to negative visual stimuli in two ways. First, it showed that among emerging adults, social regulation (via handholding with a friend) attenuates neural response in affective/salience and inhibitory control regions while viewing a set of aversive social images that elicit a broad range of negative emotions. Previous studies on social regulation of neural response have primarily focused on stimuli that elicit the specific emotion of fear and tactile rather than visual stimuli (e.g., Coan et al., 2013, 2006). Thus, the current study helps to demonstrate the neural circuitry involved when social regulation is used for contexts that elicit negative emotions more broadly. Second, the present study demonstrated that an interpersonal trait—desired emotional closeness—was associated with greater activation in response to negative social images in some regions considered key to self and social processing, somatosensory responses, and facial emotion recognition.

### 4.1 Social regulation of neural response to aversive images

The primary finding that social regulation attenuated response in a region that included areas related to affect (i.e., right dorsal striatum/anterior insula) and inhibitory control (i.e., VLPFC) is consistent with findings from previous emotion regulation studies. Most emotion regulation studies that use negative IAPS images examine cognitive emotion regulation, and they consistently find an attenuation of affective regions: the amygdala is most common, but the attenuation of the striatum and insula have also been demonstrated (Ochsner et al., 2012). Regarding the social regulation of neural response, Coan et al. (2013, 2006) found that holding a spouse's or friend's hand when anticipating an electric shock attenuated neural threat response in a myriad of regions related to affect (e.g., ventral anterior cingulate cortex, striatum), somatosensory responses (e.g., postcentral gyrus), and inhibitory control (i.e., dorsolateral prefrontal cortex; DLPFC; dorsal anterior cingulate cortex). Consistent with the present study, fewer brain regions seem to be attenuated when holding the hand of a friend (Coan et al., 2013) than a spouse (Coan et al., 2006). The attenuation of the dorsal striatum and anterior insula in a socioemotional context may be beneficial given that these regions are part of the salience network. More specifically, the dorsal striatum plays a role in habit and motor aspects of affective processing (Malvaez & Wassum, 2018) and the anterior insula is involved with the “feeling” of emotion and bodily sensations (Craig & Craig, 2009). The present finding that social regulation attenuated activation of the VLPFC is in line with a previous finding that social regulation attenuated activation of the DLPFC (Coan et al., 2006). The VLPFC and DLPFC are both considered to be key regions in neural circuitry involved in effortful emotion regulation (Phillips et al., 2008). Coan (2008) asserts that the attenuation of such regions by social regulation suggests a conservation of cognitive resources. Overall, the findings from the present and previous studies suggest consistency in the attenuation of affective and inhibitory control regions by social regulation in both nonsocial and social emotional contexts (i.e., when anticipating electric shock and when viewing images of other people in horrifying situations).

The use of electric shock and visual stimuli each provide differential benefits. The anticipation of electric shock is a specific kind of aversive context (i.e., threat) that reliably elicits a specific emotion (i.e., fear) and can easily be used across species in translational research. In contrast, a diverse set of aversive visual stimuli (e.g., IAPS) is a more general aversive context that elicits a broad range of negative emotions. This is beneficial given that social regulation occurs in various types of aversive contexts in the real world. In addition, demonstrating that social regulation of neural response can be investigated using negative images can inspire future fMRI studies that require visual stimuli. For example, using negative images as stimuli makes it easier to examine the effect of the social regulation of neural response on emotional memory. In a behavioral study, Flores and Berenbaum (2017a) found that social regulation reduces emotional memory enhancement for negative stimuli (i.e., negative IAPS images). Future studies could examine the neural circuitry involved in the impact of social regulation on long‐term memory by using IAPS images. Another benefit of using IAPS images is that it can facilitate contrasting the impact of the social regulation of neural response in negative versus positive emotional contexts (i.e., negative vs. positive images). Overall, it is advantageous to demonstrate that the neural circuitry involved in social regulation can be investigated using various types of emotional stimuli.

Previous fMRI studies on the social regulation of neural response (Coan et al., 2013, 2006) have found that both having a relationship (i.e., stranger vs. spouse or friend) and the quality of the relationship (i.e., marital quality) with the person providing a handhold matters. By explicitly comparing neural response to aversive images while merely having a friend in the room (i.e., a less active form of social regulation) versus holding that friend's hand, the present study adds the degree of physical “presence” (e.g., being in the room and holding hands vs. not holding hands) to the list of factors that alter the effectiveness of the social regulation of neural response. Handholding may strengthen the social regulation of neural response due to being a stronger signal of support than the mere presence of a person and/or through the physical sensation of touch. Future study designs should incorporate additional degrees of physical presence (e.g., absent from room) and types of physical contact, such as touching another part of the body (e.g., lower leg) that is a similar level of physical contact as handholding but may not be a strong signal of support.

### 4.2 Association between trait desired emotional closeness and neural response to negative visual stimuli

The finding that desired emotional closeness was positively associated with activation in social and somatosensory circuitry (i.e., right vPCC, right postcentral gyrus) in response to social negative images suggests further consideration of interpersonal traits in affective neuroscience. The PCC is heavily implicated in self‐processing and social cognition (i.e., right PCC; Cavanna & Trimble, 2006; Noonan et al., 2017). Compared to the dorsal PCC, the vPCC is more functionally connected with medial temporal lobe structures (e.g., hippocampus; Leech et al., 2011). The postcentral gyrus has been found to be related to action understanding (i.e., “body reading;” Van Overwalle et al., 2015), somatosensory responses (Akselrod et al., 2017), and facial emotion recognition (Hooker et al., 2012). Altogether, desired emotional closeness was associated with the activation of areas related to a variety of social cognitive functions.

The pattern exhibited by desired emotional closeness in the present study contrasts that of previous patterns found with trait loneliness. Loneliness is associated with less activation of social processing regions—namely the TPJ—when viewing negative social images compared with negative nonsocial images (Cacioppo et al., 2009). On one hand, this contrasting pattern is striking given that wanting greater interpersonal connection is integral to both desired emotional closeness and loneliness. On the other hand, this contrast is consistent with a key difference between desired emotional closeness and loneliness, such that desired emotional closeness is positively related to perceived state level of emotional closeness and negatively related to psychological distress (Flores & Berenbaum, 2014). It is possible that the neural pattern exhibited by individuals high in desired emotional closeness may be more adaptive for interpersonal connection versus the pattern exhibited by loneliness.

In addition to aversive socioemotional contexts, the present findings can encourage further examination of desired emotional closeness (or other interpersonal traits) in prosocial socioemotional processes. Given that desired emotional closeness is related to the effectiveness of social regulation in behavioral studies (Flores & Berenbaum, 2012, 2014, 2017a), a future study with a larger sample should investigate the role of desired emotional closeness in the social regulation of neural response. Future studies should also examine whether trait desired emotional closeness plays a role in neural response to social reward. Real‐world positive interpersonal interactions are related to the recruitment of social circuitry during laboratory social rewards in fMRI studies (Flores et al., 2018; Morningstar et al., 2019), as well as a reward‐sensitive event‐related potential in response to a social reward in an electroencephalogram (EEG) study (Weinberg et al., 2021). Examining the role of desired emotional closeness during other prosocial emotional processes would help to further elucidate the role of desired emotional closeness in affective neural response.

### 4.3 Limitations and other future directions

Although the present study has a modest sample size and results should be considered preliminary, we used rigorous analytical techniques (e.g., whole‐brain analyses that account for multiple comparisons) to minimize Type I error. Another limitation is that we did not find sufficient evidence that handholding attenuated subjective responses to negative stimuli. Thus, the present findings on social regulation are limited to neural responses, which is still valuable given that one of the main assertions of social baseline theory is that social regulation helps to conserve cognitive resources (e.g., neural activation) relative to most intrapersonal forms of regulation (Coan, 2008).

There are several future directions that could address other limitations of the present study, particularly those related to development, psychopathology, and measuring or manipulating cognitive emotion regulation and social processing. Although age‐related neural differences were not found in the present study, it would be beneficial to compare different developmental periods to examine whether there are developmental changes in the role of desired emotional closeness and the social regulation of neural response based on the varying importance of close relationships, intimacy of friendships, and influence of peers across development. For instance, physical touch plays a unique critical role in infant development and the formation of romantic bonds in adulthood (Field, 2019). Physical touch also has a therapeutic impact in medical/psychiatric settings (e.g., during medical procedures, agitation) and on stress, grief, and pain (Field, 2019; Kempson, 2001; Hawranik et al., 2008; Peterson et al., 2007). However, the role of physical touch and other forms of the social regulation of neural response in psychopathology remains unclear. Future studies would benefit from larger sample sizes comparing the social regulation of neural response in clinical versus nonclinical groups. The present study did not directly measure or manipulate cognitive/effortful emotion regulation and social processing. Future studies that directly measure or manipulate cognitive emotion regulation and/or social processing could be helpful.

## 5 CONCLUSION

The present study provides preliminary contributions to the current neuroscience literature on the role of interpersonal context in affective response by examining two key components of interpersonal context: (1) the presence of other people and (2) interpersonal traits. The present study builds on previous findings that social regulation attenuates neural response to a physical threat (i.e., electric shock) by demonstrating among emerging adult friends that social regulation also attenuates neural response to social visual stimuli (i.e., images of people in distressing situations) that elicit a broad range of negative emotions (including disgust, horror, and sadness). The present study also demonstrates that trait desired emotional closeness is an interpersonal trait that is associated with greater recruitment of social and somatosensory circuitry when viewing images of people in distressing situations. Thus, the present study encourages further investigation of the role of interpersonal context in neural affective response.

## CONFLICT OF INTEREST

The authors declare no conflict of interest.

### PEER REVIEW

The peer review history for this article is available at https://publons.com/publon/10.1002/brb3.2438

## Supporting information

Supporting Information

## Data Availability

Data are available from the corresponding author upon reasonable request.
