# Supplementary material for: Interpersonal context and desired emotional closeness in neural response to negative visual stimuli: Preliminary findings
Source: Brain Behav. 2021 Dec 7;12(1):e2438. doi: 10.1002/brb3.2438 (PMC8785641; doi:10.1002/brb3.2438)
Supplement: Supplementary file 1 — Supporting Information [file BRB3-12-e2438-s001.docx]

| **Neutral IAPS Images** | | |  | **Negative IAPS Images** | | |
| --- | --- | --- | --- | --- | --- | --- |
| 2020 | 2385 | 2749 |  | 2095 | 3350 | 6821 |
| 2025 | 2393 | 2780 |  | 2141 | 3500 | 6830 |
| 2038 | 2397 | 2840 |  | 2352.2 | 6021 | 6831 |
| 2104 | 2410 | 2850 |  | 2691 | 6200 | 6838 |
| 2191 | 2480 | 2870 |  | 2692 | 6212 | 8485 |
| 2200 | 2487 | 2890 |  | 2703 | 6213 | 9040 |
| 2214 | 2495 | 3550.2 |  | 2799 | 6230 | 9250 |
| 2215 | 2506 | 4571 |  | 2800 | 6242 | 9252 |
| 2220 | 2518 | 5455 |  | 2811 | 6300 | 9400 |
| 2221 | 2570 | 6570.2 |  | 3010 | 6312 | 9410 |
| 2235 | 2575 | 7044 |  | 3051 | 6313 | 9424 |
| 2280 | 2580 | 7493 |  | 3060 | 6370 | 9592 |
| 2305 | 2595 | 8232 |  | 3100 | 6510 | 9635.1 |
| 2357 | 2597 | 9070 |  | 3101 | 6540 | 9800 |
| 2372 | 2635 | 9210 |  | 3102 | 6550 | 9910 |
| 2383 | 2745.1 | 9700 |  | 3220 | 6560 | 9920 |

**Table S1**

*List of Images Included in fMRI Task from the International Affective Pictures System (IAPS)*
